# Identifying neural drivers of benign childhood epilepsy with centrotemporal spikes

**DOI:** 10.1016/j.nicl.2017.11.024

**Published:** 2017-12-05

**Authors:** Azeez Adebimpe, Emilie Bourel-Ponchel, Fabrice Wallois

**Affiliations:** a INSERM UMR 1105, Research Group on Multimodal Analysis of Brain Function, University of Picardy Jules Verne, 80036 Amiens Cedex, France; b INSERM UMR 1105, EFSN pediatric, Amiens University Hospital, Avenue Laennec, 80054 Amiens Cedex, France

**Keywords:** Benign childhood epilepsy, EEG, Dynamic causal modelling, Interictal activity

## Abstract

Epilepsy is a neurological disorder characterized by abnormal electrical discharges in a group of brain cells. Benign childhood epilepsy, which affect children under the age of 12 years, has been reported to contribute to the cognitive impairment of these children, even in the absence of structural abnormalities. Functional connectivity models have been applied to provide a deeper understanding of the processes that control and regulate interictal activity of benign childhood epilepsy. These studies have shown regions of increased connectivity and activity, particularly at the epileptic zone, which is usually the central region around the sensorimotor cortex, and in the immediate regions surrounding the zone and reduced activity in distant regions, such as the frontal lobe and temporal regions. The present study was designed to identify the neural drivers involved in the initiation and propagation of epileptic activity and the causal relationships between brain regions with increased and decreased connectivity and functional activity. We used three different models to identify neural drivers and casual connectivity with dynamic causal modelling (DCM) of EEG data. All models showed that the central region, the source of the epileptic activity, is the major driver of the brain network during interictal discharges. Other regions include the temporoparietal junction and temporal pole. The central region also had influence on the frontal and contralateral hemisphere, which might explain the cognitive deficits observed in these patients.

## Highlights

• The epileptic source is the major driver of the brain network
• Other drivers include the temporoparietal junction and temporal pole
• Epileptic source had influence on the frontal region which might explain the cognitive deficits
• The right epileptic region drives the left hemisphere during interictal epileptic discharges

## 1 Introduction

Benign childhood epilepsy (BCE) affects 10 to 20% of children with epilepsy (Camfield et al., 2014, Panayiotopoulos, 1999a, Panayiotopoulos, 1999b). The risk of cognitive impairment is higher when comparing the cognitive performance of children with BCE with that of healthy children (Danielsson and Petermann, 2009, Datta et al., 2013a, Datta et al., 2013b). Unlike adult epilepsy, such as temporal lobe epilepsy, the brain structure of BCE patients is usually normal (Fountain, 2008). However, epileptic activity can cause various malfunctions between subcortical and cortical regions that may lead to changes not only in resting state activity (Adebimpe et al., 2015a), but also in cognitive performance (Van Bogaert et al., 2012, van Rijckevorsel, 2006, Vingerhoets, 2006). The most common form of BCE is benign childhood epilepsy with central temporal spikes (BCECTS), other type of BCE include benign rolandic epilepsy and Panayiotopoulos syndrome (Panayiotopoulos, 1999a, Panayiotopoulos, 1999b). EEG is the essential diagnostic tool for BCE. The appearance of infrequent seizures or focal activity of EEG with biphasic or triphasic interictal epileptic spikes (IES) in rolandic or central brain regions is highly suggestive of benign childhood epilepsy (Bourel-Ponchel, 2013). Further analyses, including normal neurological examination and spike source imaging on high-resolution electroencephalography (HR EEG) with an anteroposterior dipole orientation, confirm the diagnosis of BCECTS (Camfield and Camfield, 2002, Panayiotopoulos, 2005).

BCECTS generally resolves by adulthood, regardless of the frequency of seizures and centrotemporal spikes (CTS), but there are concerns that BCECTS may alter both structural and functional brain properties, as the period during which CTS occur corresponds to the period of rapid brain development (Chugani et al., 1996), as demonstrated by microstructural changes of white and grey matter in the epileptic zone (Kim et al., 2014) and disturbances of grey matter growth in frontal and insular regions (Kanemura and Aihara, 2009, Pardoe et al., 2013). It should be noted that these regions are involved in language and attention processing. Other studies have reported reduced structural and functional connectivity activities, which might delay structural and functional brain development (Besseling et al., 2013a, Kim et al., 2014). Children with BCECTS are reported to perform poorly compared to healthy controls, especially in visuospatial and verbal fluency tests, language and hearing (Besseling et al., 2013b), memory (Lopes et al., 2014) and behavioural problems, such as more aggressive behaviour, social problems, depression and attention deficits (Dunn, 2014, Pąchalska et al., 2012). Antiepileptic drugs (AEDs) might reduce CTS by suppressing the amplitude of the spikes, but some studies have indicated that some AEDs might worsen language and cognitive functions, raising a concern about the trade-off of benefits and risks related to AEDs (Camfield and Camfield, 2002, Park and Kwon, 2008). A few studies have reported that some deficits can persist throughout adulthood, even when the patients no longer experience BCECTS (Camfield and Camfield, 2002). Considering these altered functional properties, EEG studies on BCECTS have reported that patients present increased delta and theta power and increased synchronization, which can be related to the disorganization of electrical activity related to epileptic activities occurring during brain development (Adebimpe et al., 2015a, Adebimpe et al., 2015b).

A large number of studies have tried to assess the functional connectivity pattern of BCECTS, especially in comparison with healthy controls (Adebimpe et al., 2016, Adebimpe et al., 2015b, Besseling et al., 2013b). The brain network of these patients has been reported to be disrupted. In particular, reduced connectivity in the default mode network, increased functional connectivity in the sensorimotor region and abnormal functional connectivity between language network and frontal regions have been reported (Adebimpe et al., 2015b, Clemens, 2004, Clemens et al., 2016, Oser et al., 2014). EEG functional connectivity studies have also reported higher theta synchronization, notably during epileptic activity and decreased alpha and beta functional connectivity in the occipital regions (Adebimpe et al., 2015b, Clemens et al., 2016). However, a better understanding of the directionality of connectivity is essential to determine whether epileptic regions have a direct or indirect influence on other distant regions, especially those related to language and cognitive networks.

To study these aspects, the dynamic causal modelling (DCM) (Kiebel et al., 2008) was applied as a measure of effective connectivity, to accurately track and quantify CTS dynamics and its impact on certain selected regions of interest (ROI). Dynamic causal modelling (DCM) is an established procedure for the analysis of both functional magnetic resonance imaging (fMRI) and electrophysiological recordings (Friston et al., 2003) and provides a generative spatiotemporal model for EEG and MEG responses with dynamic input and output (David et al., 2006). DCM is a Bayesian model scheme with competing hypotheses that identifies directional connectivity patterns and connection strengths of neuronal activity. DCM has been used to study neural drivers and to identify epileptic foci of IES with both EEG/MEG and fMRI; and with simultaneous EEG-fMRI recordings (Murta et al., 2012). More specifically, it has been used to study the seizure activity with EEG and ECOG (Cooray et al., 2016, Papadopoulou et al., 2017).

The primary objective of this study using DCM on scalp HR EEG data was to investigate the main neural drivers and causal relationships or coupling between identified interictal epileptic region of BCECTS patients and other distant ROI that have been reported to be affected by the presence of IES by previous studies from our laboratory (Adebimpe et al., 2015a, Adebimpe et al., 2015b, Bourel-Ponchel et al., 2017) and the literature (Clemens, 2004, Clemens et al., 2010, Yeom et al., 2014).

## 2 Methods

### 2.1 Data

This study was conducted in 12 BCECTS patients (age: 9.38 ± 2.39 years, 5 females) with right centrotemporal spikes. All patients had IES in the right hemisphere. Patient selection was based on criteria concerning common source location at the central region, anteroposterior dipole orientation (Camfield and Camfield, 2002), similar interictal epileptic patterns and no evidence of any structural brain damage based on magnetic resonance imaging (MRI). BCECTS was diagnosed on the basis of a typical clinical history and the presence of characteristic IES on standard EEG, according to ILAE criteria (Berg et al., 2010). Clinical diagnostic criteria of BCECTS included children presenting sensorimotor seizures with inconsistent secondary generalization, with an age of onset between 4 and 10 years (Beaumanoir et al., 1974) and typical diphasic spikes either isolated or occurring in clusters, unilaterally or bilaterally, in the centro-temporal areas on a standard normal background EEG (Beaumanoir et al., 1974). Patients with an abnormal neonatal history, intellectual deficit (IQ < 70), neurological abnormalities on physical examination, and/or any lesions in brain neuroimaging were not included in the study.

**Fig. 1:**
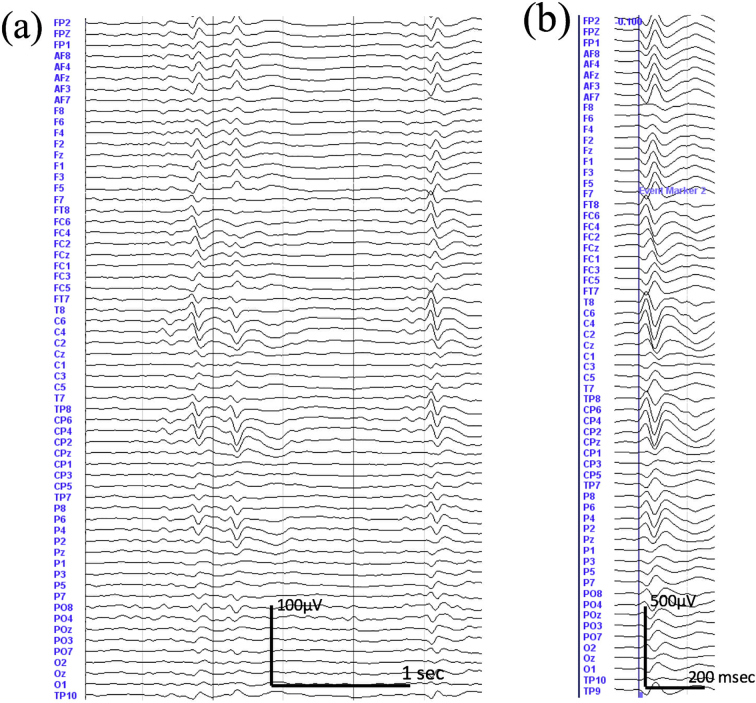
Patient EEG. (A) Raw data of one of the patients filtered between 0.5 and 15 Hz with interictal epileptic spikes (IES) in the right hemisphere electrodes (C4, C6, T8, CP4, CP6 and TP8). (B) The grand average of 50 IES showed high amplitude spike activity in the right hemisphere electrodes.

To define a homogeneous sample of patients for both single subject and group analyses, twelve patients with right centro-temporal spikes have been selected. This includes two patients with bilateral IES occurring independently from one to the other hemisphere. Table 1 lists the patient's clinical characteristics. Fig. 1 provides the EEG sample of one patients.

**Table 1:** Patient's clinical characteristics.

patient number	Age (years)	Neuropsychological data	Seizure free	Interictal EEG	Treatment at time of HD EEG
1	12.63	Normal	No	Unilateral IES	Valproate sodium
2	12.64	Normal	Yes	Unilateral IES	Valproate sodium
3	9.25	Attention deficit	No	Unilateral IES	Oxcarbazepine
4	6.03	Normal	No	Unilateral IES	Oxcarbazepine
5	10.47	Attention deficit	Yes	Unilateral IES	Valproate sodium
6	7.16	Normal	Yes	Unilateral IES	Valproate sodium
7	8.51	Attention deficit	Yes	Unilateral IES	No
8	13.16	Normal	Yes	Bilateral^a^ independent IES	Valproate sodium
9	9.67	Language Deficit	Yes	Unilateral IES	Lamotrigine
10	7.79	Language deficit	Yes	Unilateral IES	Oxcarbazepine
11	8.2	Normal	Yes	Bilateral^a^ independent IES	No
12	7.1	Attention deficit	Yes	Unilateral IES	Valproate sodium

a Bilateral: patients with bilateral IES occurring independently from one to the other hemisphere.

### 2.2 Ethical considerations

The study was approved by the local ethics committee (CPP Nord-Ouest No. A00782-39) Written informed consent to participate in the study was obtained from the parents and all patients before inclusion.

### 2.3 EEG recordings and pre-processing

All patients underwent at least a 14-minute 64-channel EEG recording (ANT, Netherlands) with electrodes placed on the scalp in accordance with the international 10-10 system (EasyCap®) at 512 Hz sampling rate. Only a notch filter (50 Hz) was applied. A mastoid reference was used for acquisition. HD EEG recordings were performed during quiet arousal. The electrode impedances were kept below 5 kΩ. The signals were re-referenced to an average reference for further analysis. Patients were monitored for movements during acquisition to allow subsequent exclusion of altered data.

### 2.4 EEG spike selection and data pre-processing

For IES selection, artefact rejection and all subsequent analyses, data were arithmetically re-referenced to an average reference. A bandpass filter between 1 and 70 Hz was applied to the continuous recording before review by the electrophysiology experts (FW, EB) who independently identified the IES. Fifty IES were selected for each patient. Typical BCECTS IES were characterized by diphasic or triphasic patterns distributed in the centrotemporal areas. Non-overlapping epochs lasting 4000 ms centered on the IES were considered for each IES. A few channels showing high impedance or artefact were interpolated by spline interpolation (Perrin et al., 1989) and portions with a majority of electrodes with artefacts were neglected and rejected. EEG were then exported for further analysis offline. The EEG was filtered offline between 1 and 40 Hz. Independent component analysis (ICA) step was applied to separate EEG activities from other hidden artefactual data such as eye blinking, cardiac and muscle artefacts (Delorme et al., 2012, Jung et al., 2000).

### 2.5 Source analysis

EEG source localization to identify the sites of IES was performed with eLORETA (exact Low-Resolution Electromagnetic Tomography), which models 3D distributions of EEG cortical sources (Pascual-Marqui et al., 2002) The eLORETA algorithm produces current density (current intensity/area, measured in A/m^2^) for each voxel. Results were normalized for each patient before computing the grand average.

### 2.6 Definition of ROIs

**Fig. 2:**
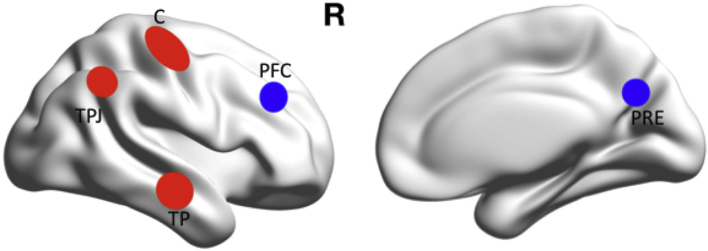
Regions of interest (ROIs). Increased source activity at the epileptic foci in the central (C) region has been reported in the literature and this increased activity extended to the ipsilateral temporo-parietal junction (TPJ) and temporal pole (TP) of the epileptic foci. However, lower activity was reported at the frontal region (prefrontal cortex – PFC), very close to the epileptic foci, and at the precuneus (PRE).

Based on our previous studies (Adebimpe et al., 2015a, Adebimpe et al., 2015b, Bourel-Ponchel et al., 2017) and the related literature (Clemens, 2004, Clemens et al., 2010, Yeom et al., 2014), five ROIs were defined to investigate the causal influence from epileptic zones to distant ROIs (Fig. 2 and Table 2). The ROIs included the central epileptic region (rC), the temporo-parietal junction (TPJ) and the temporal pole (TP), which always produced intense activity in the presence of IESs Other ROIs included the ipsilateral prefrontal cortex (PFC) and the precuneus (PRE), which usually presented decreased activity during IES (Adebimpe et al., 2015a, Bourel-Ponchel et al., 2017, Clemens et al., 2007). The central epileptic region (C) was identified on high-resolution MRI with common high source activity. Other ROIs were identified from the AAL atlas (Tzourio-Mazoyer et al., 2002).

**Table 2:** Regions of interest (ROIs) coordinates including epileptic foci. The locations are based on Montreal Neurology Institute (MNI) space (in mm).

Right central (rC)- epileptic foci	44, − 15, 41
Left central (lC)	− 44, − 15, 41
Right prefrontal cortex (rPFC)	38, 34, 24
Left prefrontal cortex (lPFC)	− 38, 34, 24
Right temporal pole (rTP)	52, 2, − 28
Right temporo-parietal junction (rTPJ)	56, − 53, 27
Right precuneus (rPRE)	11, − 55, 47

### 2.7 DCM analysis

**Fig. 3:**
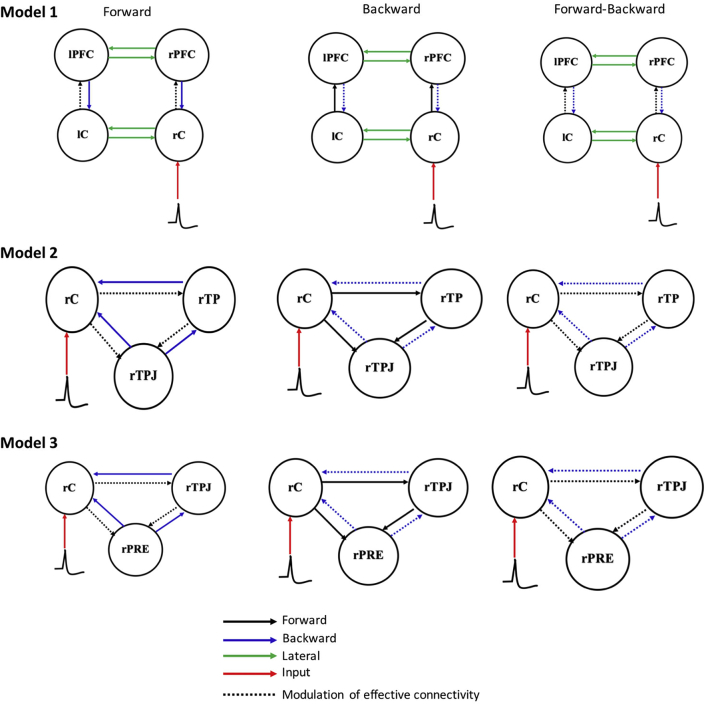
Model specifications. Three models were composed of three sub-models: forward (F), backward (B) and forward-backward (FB) models. The sources comprised the epileptic zone at the right central (rC), left central (lC), right temporal pole (rTP), right temporo-parietal junction (rTPJ), right and left prefrontal cortex (rPFC, lPFC) and right precuneus (rPRE). There are forward (black), backward (blue) and lateral (green) connections and the dotted line indicates the modulation of effective connectivity, i.e. changes in connection. (For interpretation of the references to color in this figure legend, the reader is referred to the web version of this article.)

DCM analysis was performed with DCM12 module as implemented in SPM12. To evaluate the causal influence between the central epileptic zone and other distant ROIs, three different models (Fig. 3) were defined based on our previous results (Adebimpe et al., 2015a, Adebimpe et al., 2015b, Bourel-Ponchel et al., 2017). Due to presume differences in the subjects despite our critical assessment of EEG profile and localization of IES activity, we decided to make each model very simple because DCM output relates to nonlinear correlates of the source activity. As one of the simple rules of DCM (Stephan et al., 2010), increasing number of ROIs with one input is practically impossible to achieve convergence across all the subjects. Therefore, we make each model simple in order to reach convergence across the subjects. For each model, the modulation of effective connectivity was investigated for the forward (F), backward (B) and forward-backward (FB) models. In all models, the right central epileptic zone (rC) was designated as the input of the neuronal activity. In the first model, we hypothesized that during IES, the rC may drive the right dorsolateral prefrontal cortex (rPFC), while the left central cortex (lC) may provide a compensatory mechanism for normal brain function in the other hemisphere. The second model investigated causal influence between the three regions (rC, rTPJ and rTP) that are always activated during IES. In the last model, we investigated whether the presence of IES had a direct influence on the decreased activity in the PRE-or an indirect influence via the TPJ.

The grand average data (50 segments) were bandpass filtered (1–30 Hz) and windowed (0–400 ms around IES activity). To map the cortical activity, we used a lead field based on the standard MRI template and a boundary element model as implemented in SPM12. To visually compare the measured/estimated data and to judge the fit of the modelling we quantified the evoked responses within the defined window after the source reconstruction. The sources and ROIs were modeled with the vertices of these sources in the same lead fields as used for the source reconstruction.

The inference on each model was performed by the Fixed effect Bayesian Model selection (FFX BMX). Each model was compared individually for each patient and optimal model parameters were obtained across all subjects with FFX and Bayesian Parameters Averaging (BPA). We examined the modulation of effective connectivity for F, B and FB models and the directionality between pairs or regions of interest (rC to lC, lC to rC, rC to rPFC, etc.). The DCM results were evaluated on the basis of the relative log evidence, posterior probability and average coupling gain to measure the effectiveness of the model and the coupling strength between brain regions.

## 3 Results

**Fig. 4:**
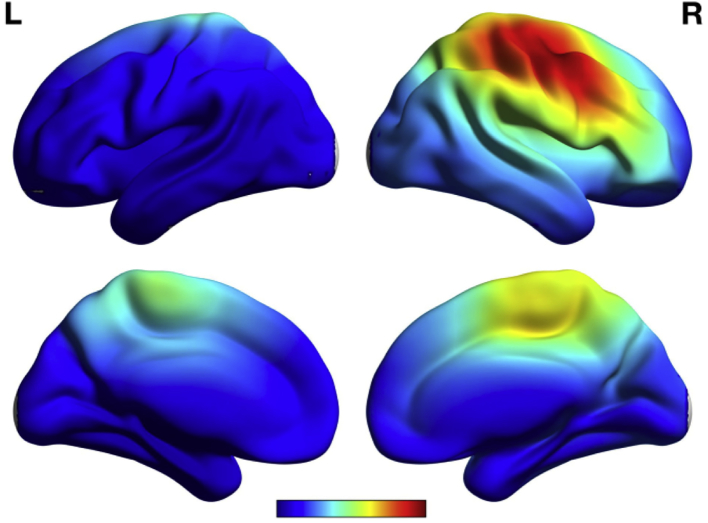
Electrical source imaging. Average current density distribution across patients shows higher activity at the central region of the epileptic zone. Source imaging was estimated for signals for 200 ms before and after the spike.

EEG source imaging performed with eLORETA from EEG segments (200 ms before and after the spike (after alignment and averaging across the epochs for each subject) indicated high source activity, during IES, covering the right central region only (Fig. 4).

### 3.1 DCM analysis

**Fig. 5:**
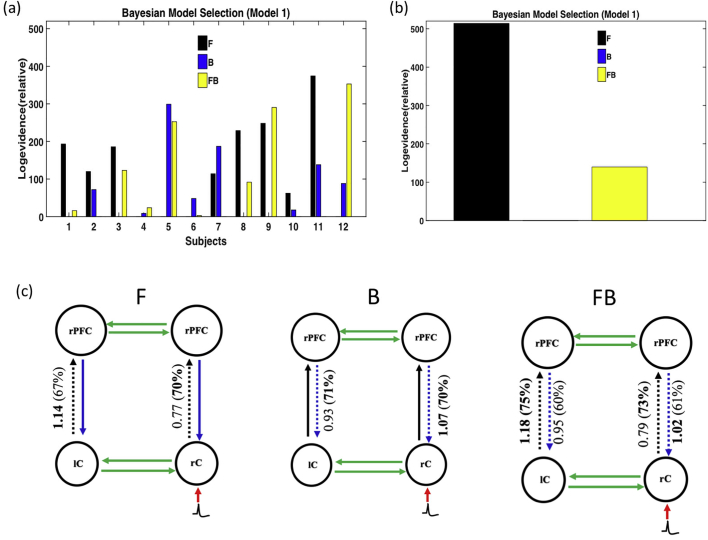
Model 1 DCM results. (a) Relative log evidence of the forward (F), backward (B) and forward-backward (FB) models for each subject compared to the null model and (b) presents family relative log evidence for all subjects, showing that the forward model is markedly superior to the other two models. Panel (c) shows the average coupling gains and their corresponding posterior probability (in brackets).

As shown in Fig. 3, three DCM models were used and each model with three forward (F) only, backward (B) only and forward-backward (FB) submodels. In Model 1, the relative log evidence was higher in the F-model than in the other 2 models for 7 out of 12 subjects (Fig. 5a). The relative log evidence was higher in the FB model in only two subjects. At the group level, the relative log evidences were higher in both the F and FB models, suggesting that they are clearly better than the B model. This was particularly true for the log evidence of the F model, which was much stronger than for the FB model. Fig. 5c shows the average coupling gains and posterior probability for model 1.

A 1.14 coupling gain in the F model between the left central (lC) and the left dorsolateral prefrontal cortex (lPFC) corresponds to an increase in effective connectivity (+ 14%) between lC and lPFC. A 0.77 coupling gain in the F model between the right central (rC) and the right dorsolateral prefrontal cortex (rPFC) corresponds to a decrease in effective connectivity (− 23%) between rC and rPFC. A 0.93 and 0.95 coupling gain in the B and FB models between lC and lPFC correspond to a decrease in effective connectivity (7 and 5%, respectively) between lC and lPFC. A 1.07 and 1.02 coupling gain in the B and FB models between rC and rPFC corresponds to an increase in effective connectivity (7 and 2%, respectively) between rC and rPFC.

**Fig. 6:**
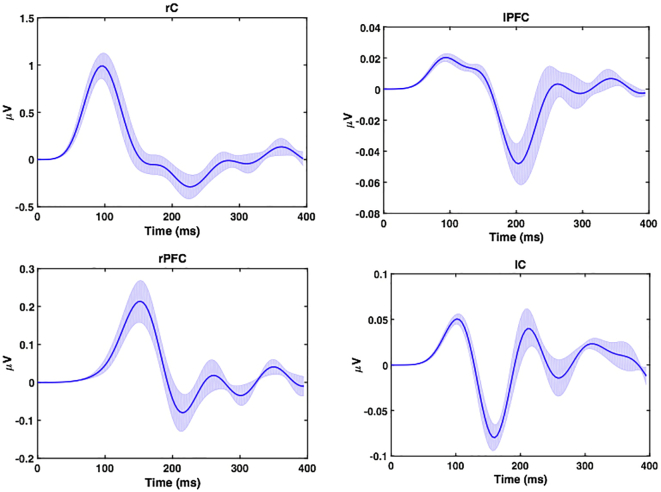
Reconstructed source activity for Model 1 (Fig. 5C). Source activity reconstructed for the different ROIs (rC), lC, rPFC and lPFC. The solid line corresponds the mean while the grey line corresponds to the standard error across the subjects.

Altogether, the F model develops a much stronger log evidence, suggesting that the forward interaction from rC to rPFC is prominent. As shown in Table 3, for bilateral connections between central (rC – lC) and frontal (rPFC - lPFC) regions, the right hemisphere is likely to drive the left hemisphere during IES in all models. The reconstructed source activities (Fig. 6) reflect both intrinsic and recurrent interactions among different neuronal pyramidal populations. The evoked successive responses peak first at about 100 ms in rC (with higher amplitude) and then, with a 50 ms delay, at about 150 ms in rPFC.

**Table 3:**
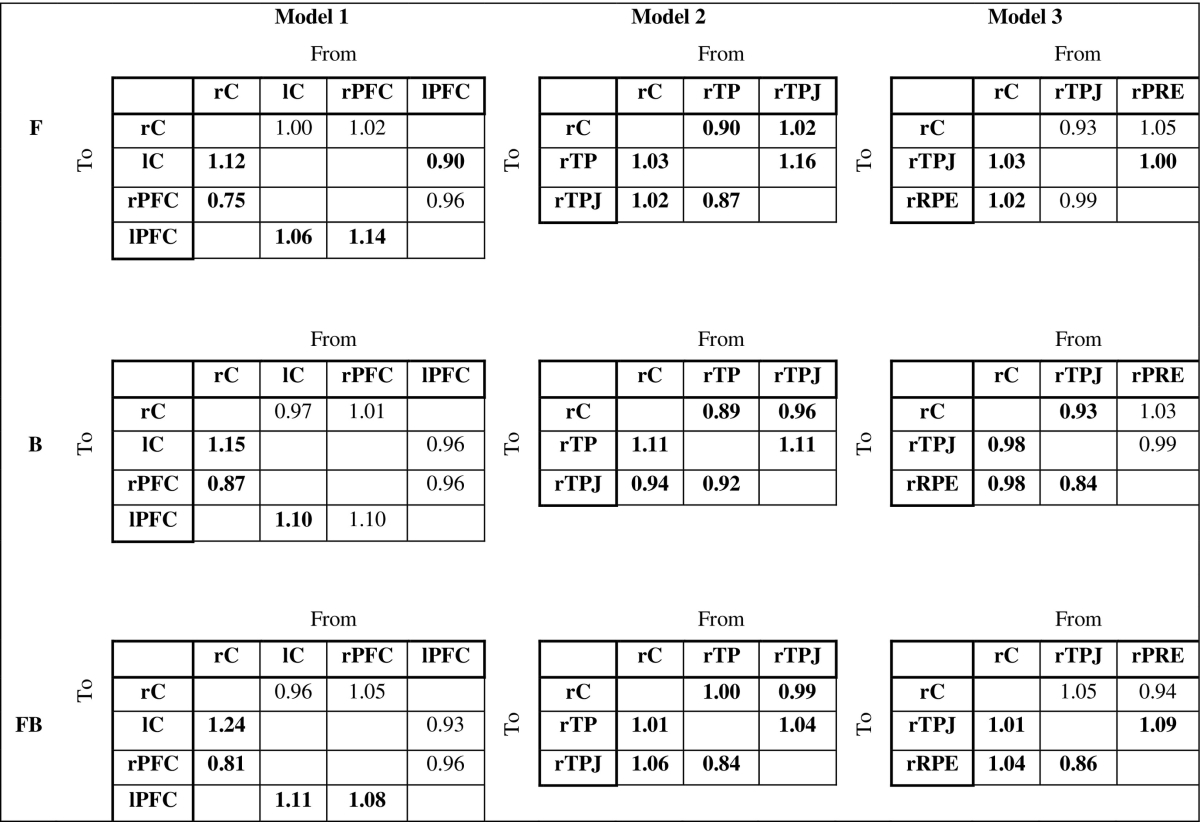
Connections strength between the sources in all three models and the corresponding forward (F), backward (B) and forward-backward (FB) submodels. The connection strength with posterior probability > 70% are shown in bold. Full table with posterior probability is shown in Supplementary table. Connections strength between the sources in all three models and the corresponding forward (F), backward (B) and forward-backward (FB) submodels. The connection strength with posterior probability > 70% are shown in bold. Full table with posterior probability is shown in Supplementary table.

**Fig. 7:**
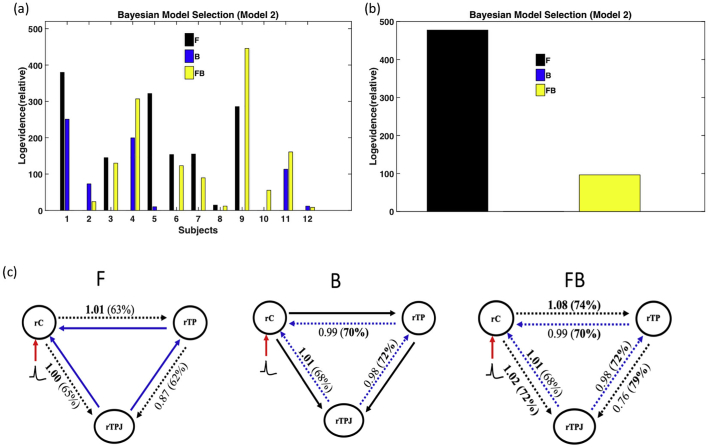
Model 2 DCM results. (a) Relative log evidence of the forward (F), backward (B) and forward-backward (FB) models for each subject compared to the null model and (b) presents family relative log evidence for all subjects, which shows that the forward model is markedly superior compared to the other two models. Panel (c) shows the average coupling gains and their corresponding posterior probability (in brackets).

Similar trends were observed in model 2 (Fig. 7a). Six subjects had higher relative log evidence in the F model and four subjects had higher relative log evidence in the FB model. Comparison across subjects (Fig. 7b) showed that, as in model 1, the F model had a higher relative log evidence compared to the FB and B models Fig. 7c presents the connectivity and coupling gain between the three ROIs for the three models.

**Fig. 8:**
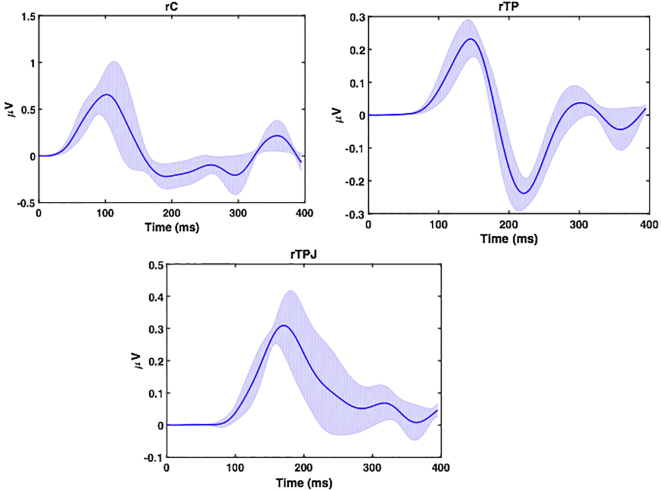
Reconstructed source activity for Model 2 (Fig. 7C). Reconstructed source activity for the different ROIs (rC), rTP, and rTPJ. The solid line corresponds the mean while the grey line corresponds to the standard error across the subjects.

The coupling gain increased in the F and FB models between the right central cortex (rC) and the right temporal pole (rTP) (1.01 and 1.08) and between the right central cortex (rC) and the temporal pole junction (rTPJ) (1 and 1.02), corresponding to an increase in effective connectivity between rC and rTP and between rC and rTPJ. The coupling gain decreased between rTPJ and rTP (with strong evidence of posterior probability (> 70%)) in all three models, notably in the FB model). Altogether these results suggest that the central epileptic zone rC drives both the rTP and rTPJ and that there are mutual causal gains between rC and rTPJ in the FB model. Similarly, reconstructed source activities (Fig. 8) showed that evoked responses peaked at about 100 ms in rC and with a 50 ms delay at about 150 ms in the two other ROIs-rTP and rTPJ.

**Fig. 9:**
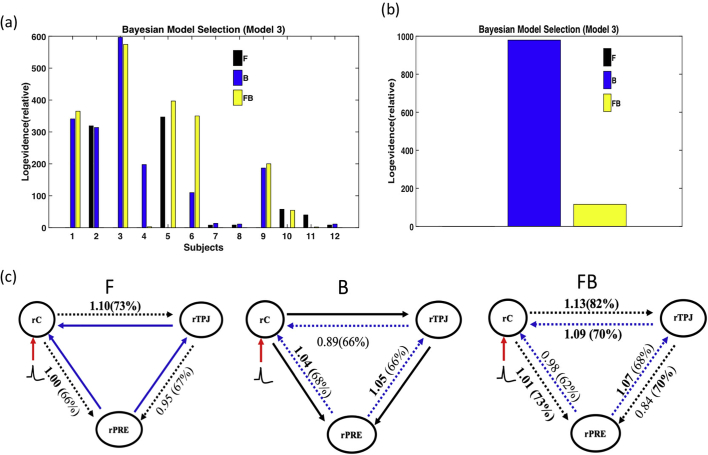
Model 3 DCM results. (a) Relative log evidence of the forward (F), backward (B) and forward-backward (FB) models for each subject compared to the null model and (b) presents family relative log evidence for all subjects, which shows that the forward model is markedly superior compared to the other two models. Panel (c) shows the average coupling gains and their corresponding posterior probability (in brackets).

The results for relative log evidence for model 3 are fairly different from those of models 1 and 2. The B and FB models were superior in terms of log evidence for 5 subjects and 4 subjects, respectively (Fig. 9a). At the group level, the B model had the highest relative log evidence and therefore constituted the best model (Fig. 9).

Fig. 9c presents the connectivity and coupling gain between the three ROIs for the three models. Coupling gain still increased in the F and FB models between the right central cortex (rC) and the right temporal pole junction (rTPJ) (1.1 and a 1.13) and between the right central cortex (rC) and the right precuneus (rPRE) (1 and 1.01), corresponding to increased effective connectivity between rC and rTPJ and between rC and rPRE in both the F and FB models. In the B model and FB model, coupling gain increased between rPRE and rTPJ (1.05 and 1.07) and between rPRE and rC (1.04 and 1.01), which could correspond to an increase in effective connectivity between rPRE and rTPJ and between rPRE and rC in both the B and FB models.

Altogether, these results suggest that the central epileptic zone (rC) drives both the rPRE and the rTPJ with direct mutual interactions between rC and rPRE in the F model. In addition, in the B and FB models, rPRE would drive rC and rTPJ, suggesting that the predicted influence from rC to rPRE may not be direct. Mutual interaction between rC and rTPJ was observed in the FB model, as in model 2.

**Fig. 10:**
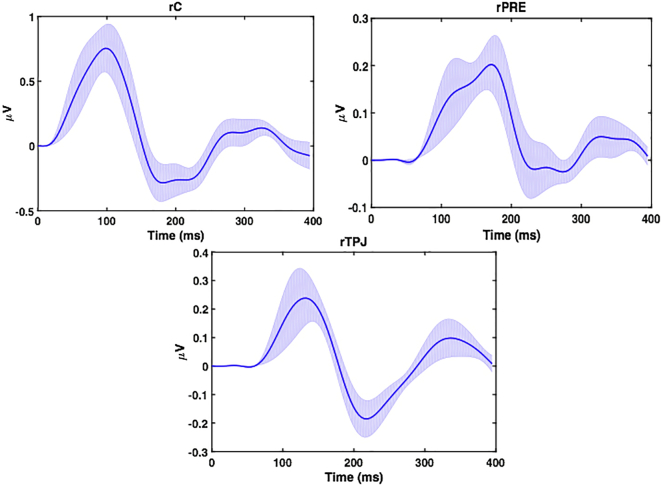
Reconstructed source activity for Model 3 (Fig. 9C). Reconstructed source activity for the different ROIs (rC), rPRE, and rTPJ. The solid line corresponds the mean while the grey line corresponds to the standard error across the subjects.

The maximum amplitude of the reconstructed source activities (Fig. 10) peaked at about 100 ms after IES in rC and after a delay of around 50 ms at about 150 ms in rTPJ and rPRE. The peak response differences between the sources (rC) and rTPJ and rPRE correspond to the effect of IES despite the fact that backward model had a higher relative log evidence (Fig. 9a and b).

In summary, the right central epileptic region drives the left hemisphere during IES, with a more marked influence in the left hemisphere than in the right hemisphere from the central region to the frontal region. In addition, differences in coupling gain were observed between the right and left hemispheres (rC to rPFC vs lC to lPFC, p = 0.0183). Based on the winning models in models 2 and 3, the right central region (rC) drives both the ipsilateral temporal pole (rTP) and temporo-parietal junction (rTPJ), with a direct mutual interaction between rC and rTPJ. The major influence of rC on the brain network organization was also evidenced from the interaction with the rPRE in the B and FB models.

## 4 Discussion

DCM analysis of BCECTS EEG data identified causal links from the right central zone of the epileptic zone to the prefrontal cortex (PFC), right temporo-parietal junction (TPJ) and temporal pole (TP), confirming that the right central zone constitutes the original key area of IES propagation in BCECTS. The present results are consistent with our previous power spectrum analysis (Bourel-Ponchel et al., 2017) and source analysis (Adebimpe et al., 2015a, Yeom et al., 2014). The main advantage of DCM analysis as an effective functional connectivity method is that it is able to detect the connectivity interaction and identify the epileptic focus based on comparison of competing connectivity models with different neural drivers. All these analyses could provide a meaningful tool to evaluate the network alterations induced by IES in BCECTS and to investigate the pathophysiology of the cognitive impact of these disorganizations (Danielsson and Petermann, 2009, Datta et al., 2013a, Datta et al., 2013b).

Higher source activity and specific scalp EEG power spectrum changes (Adebimpe et al., 2015a, Bourel-Ponchel et al., 2017) in the ipsilateral central and frontal regions of the epileptic foci suggested the involvement of a frontocentral network during IES. As expected, the central epileptic region drives or exerted a greater causal influence on the frontal region, suggesting the involvement of the frontal regions as one of the main sinks of epileptic activity. Also, peak response differences between the right central and prefrontal region can be interpreted as the effect of the right central on the prefrontal regions which support the winning of the Forward model. The lateral hemisphere (lC and LPFC) show different pattern of source responses which can be due to a lesser involvement of these regions during the IES or of the occurrence of a compensatory mechanism in distant bran region. However, the causal link between the central and frontal or frontocentral network acts as a source of abnormal information flow onto the frontal areas during IES in BCECTS and suggests that IES may play a role in the alteration of the attention network (Kaufmann et al., 2009). In other words, the ipsilateral frontal cortex receives a causal effect driven by the central epileptic region. The dorsolateral prefrontal cortex (PFC) is a region of the frontal lobes that is most typically associated with executive functions, including working memory and selective attention (Curtis and D'Esposito, 2003), conscious decision making, reasoning, working memory, inhibition, as well as outcome prediction (Krawczyk, 2002). It is also a key node in attention networks that support basic cognitive selection of sensory information and response (Corbetta and Shulman, 2002) and all of these functions are important in complex cognitive tasks such as learning, language and cognitive evaluation (Badre et al., 2009). This might support the evidence of frontal region growth disturbance (Kanemura and Aihara, 2009) and has been correlated with the attention and cognitive deficits in BCECTS patients (Dunn, 2014, Lopes et al., 2014) together with the alteration of white matter microstructure of the BCECTS brain (Kim et al., 2014). This finding can be considered to support the results of several fMRI and MEG/EEG studies indicating the role of the frontal cortex in the initiation and propagation of IES (Panzica et al., 2013, Wu et al., 2015).

Because, the mutual interaction increased between the left central and the left frontal regions, it supposes that the right central epileptic foci may also influence the contralateral hemisphere. This may correspond to a reorganization of the brain network during IES serving as a compensatory mechanism in the contralateral hemisphere (Datta et al., 2013a, Datta et al., 2013b). Altogether, whatever the ipsilateral or contralateral engagement of a frontocentral network, our results are consistent with an influence from the central IES region on the cognitive abilities of BCECTS patients (Li et al., 2015).

The causal influence of the right central regions to the temporal pole (TP) and temporoparietal junction (TPJ) support evidence that impairment of the BCECTS brain network is not restricted to the epileptogenic focus or the frontocentral pathway. The TPJ is a region involved in social interactions and mentalizing (Mizuguchi et al., 2016). The influence of epileptic activity on this region (TPJ) may also have an effect on cognitive and learning performance in BCECTS patients (Hewett et al., 2011, Mosher et al., 1992). The similar pattern of the of evoked responses of the reconstructed source activities in rC and rTPJ and rTP support their possible involvement during IES but the peak response differences support the evidence that the rC is major driver of the epileptic network. The driving or causal influence from the central zone to the temporal pole (TP) suggests possible impairment of other brain networks, such as those involved in face recognition, auditory, visual and language networks (Bonner and Price, 2013, Olson et al., 2007), supporting the high prevalence of language impairment in children with BCECTS (Besseling et al., 2013a, Piccirilli et al., 1988, Wolff et al., 2005). Interestingly, many patients with BCECTS have a specific language-related learning disorder (not a general learning disorder). The causal influence from the central region to the temporal pole observed in this study supports the reported correlation between impaired motor development and language impairment in children with BCECTS (Besseling et al., 2013a, Besseling et al., 2013b). To thoroughly investigate the altered language network in patients with BECTS especially the suspected direct influence of the epileptic zone to Broca's area, a complementary study of both the left and right centrotemporal areas need to be investigated. This is very important due to dominant language network in the left hemisphere.

In conclusion, the central region, the source of the epileptic activity, is the major driver of the brain network during IES. The causal influence from the central to the ipsilateral frontal region, temporal, temporoparietal regions and to the contralateral hemisphere suggests that the BCECTS brain network is widely altered during IES. These results provide new insight into the cognitive deficits reported in children with BCECTS.

The following is the supplementary data related to this article.

Table S1 Posterior estimates and probabilities for the connections strength between the sources all the three models and the corresponding forward (F), backward(B) and forward-backward(FB) sub models. Supplementary table
