# Supplementary material for: Identifying neural drivers of benign childhood epilepsy with centrotemporal spikes
Source: Neuroimage Clin. 2017 Dec 5;17:739–50. doi: 10.1016/j.nicl.2017.11.024 (PMC5730126; doi:10.1016/j.nicl.2017.11.024)
Supplement: Table S1 — Posterior estimates and probabilities for the connections strength between the sources all the three models and the corresponding forward (F), backward(B) and forward-backward(FB) sub models. [file mmc1.docx]

|  |  |  |  |  | **Model 1** | | | |  |  |  |  |  |  |
| --- | --- | --- | --- | --- | --- | --- | --- | --- | --- | --- | --- | --- | --- | --- |
|  |  |  | **Posterior estimates** | | |  |  |  |  | **Posterior probablities** | | |  |  |
|  |  |  |  | From | |  |  |  |  |  | From | |  |  |
|  |  |  | **rC** | **lC** | **rPFC** | **lPFC** |  |  |  | **rC** | **lC** | **rPFC** | **lPFC** |  |
|  |  | **rC** |  | 1.00 | 1.02 |  |  |  | **rC** |  | 0.62 | 0.64 |  |  |
| **F** | To | **lC** | 1.12 |  |  | 0.90 |  | To | **lC** | 0.77 |  |  | 0.72 |  |
|  |  | **rPFC** | 0.75 |  |  | 0.96 |  |  | **rPFC** | 0.83 |  |  | 0.67 |  |
|  |  | **lPFC** |  | 1.06 | 1.14 |  |  |  | **lPFC** |  | 0.74 | 0.76 |  |  |
|  |  |  |  |  |  |  |  |  |  |  |  |  |  |  |
|  |  |  |  |  |  |  |  |  |  |  |  |  |  |  |
|  |  |  |  | From | |  |  |  |  |  | From | |  |  |
|  |  |  | **rC** | **lC** | **rPFC** | **lPFC** |  |  |  | **rC** | **lC** | **rPFC** | **lPFC** |  |
|  |  | **rC** |  | 0.97 | 1.01 |  |  |  | **rC** |  | 0.64 | 0.69 |  |  |
| **B** | To | **lC** | 1.15 |  |  | 0.96 |  | To | **lC** | 0.77 |  |  | 0.69 |  |
|  |  | **rPFC** | 0.87 |  |  | 0.96 |  |  | **rPFC** | 0.82 |  |  | 0.63 |  |
|  |  | **lPFC** |  | 1.10 | 1.10 |  |  |  | **lPFC** |  | 0.71 | 0.66 |  |  |
|  |  |  |  |  |  |  |  |  |  |  |  |  |  |  |
|  |  |  |  |  |  |  |  |  |  |  |  |  |  |  |
|  |  |  |  | From | |  |  |  |  |  | From | |  |  |
|  |  |  | **rC** | **lC** | **rPFC** | **lPFC** |  |  |  | **rC** | **lC** | **rPFC** | **lPFC** |  |
|  | To | **rC** |  | 0.96 | 1.05 |  |  |  | **rC** |  | 0.60 | 0.66 |  |  |
| **FB** |  | **lC** | 1.24 |  |  | 0.93 |  | To | **lC** | 0.79 |  |  | 0.66 |  |
|  |  | **rPFC** | 0.81 |  |  | 0.96 |  |  | **rPFC** | 0.78 |  |  | 0.61 |  |
|  |  | **lPFC** |  | 1.11 | 1.08 |  |  |  | **lPFC** |  | 0.72 | 0.72 |  |  |
|  |  |  |  |  |  |  |  |  |  |  |  |  |  |  |
|  |  |  |  |  |  |  |  |  |  |  |  |  |  |  |
|  |  |  |  |  |  | **Model 2** | | | |  |  |  |  |  |
|  |  |  | **Posterior estimates** | | |  |  |  |  | **Posterior probablities** | | |  |  |
|  |  |  |  | From |  |  |  |  |  | From |  |  |  |  |
|  |  |  | **rC** | **rTP** | **rTPJ** |  |  |  |  | **rC** | **rTP** | **rTPJ** |  |  |
|  |  | **rC** |  | 0.90 | 1.02 |  |  |  | **rC** |  | 0.73 | 0.70 |  |  |
| **F** | To | **rTP** | 1.03 |  | 1.16 |  |  | To | **rTP** | 0.74 |  | 0.74 |  |  |
|  |  | **rTPJ** | 1.02 | 0.87 |  |  |  |  | **rTPJ** | 0.71 | 0.74 |  |  |  |
|  |  |  |  |  |  |  |  |  |  |  |  |  |  |  |
|  |  |  |  |  |  |  |  |  |  |  |  |  |  |  |
|  |  |  |  |  |  |  |  |  |  |  | From |  |  |  |
|  |  |  | **rC** | **rTP** | **rTPJ** |  |  |  |  | **rC** | **rTP** | **rTPJ** |  |  |
|  |  | **rC** |  | 0.89 | 0.96 |  |  |  | **rC** |  | 0.74 | 0.69 |  |  |
| **B** | To | **rTP** | 1.11 |  | 1.11 |  |  | To | **rTP** | 0.74 |  | 0.70 |  |  |
|  |  | **rTPJ** | 0.94 | 0.92 |  |  |  |  | **rTPJ** | 0.75 | 0.69 |  |  |  |
|  |  |  |  |  |  |  |  |  |  |  |  |  |  |  |
|  |  |  |  |  |  |  |  |  |  |  |  |  |  |  |
|  |  |  |  | From |  |  |  |  |  |  | From |  |  |  |
|  |  |  | **rC** | **rTP** | **rTPJ** |  |  |  |  | **rC** | **rTP** | **rTPJ** |  |  |
|  |  | **rC** |  | 1.00 | 0.99 |  |  |  | **rC** |  | 0.76 | 0.73 |  |  |
| **FB** | To | **rTP** | 1.01 |  | 1.04 |  |  | To | **rTP** | 0.72 |  | 0.73 |  |  |
|  |  | **rTPJ** | 1.06 | 0.84 |  |  |  |  | **rTPJ** | 0.67 | 0.80 |  |  |  |
|  |  |  |  |  |  |  |  |  |  |  |  |  |  |  |
|  |  |  |  |  |  |  |  |  |  |  |  |  |  |  |
|  |  |  |  |  |  | **Model 3** | | | |  |  |  |  |  |
|  |  |  | **Posterior estimates** | | |  |  |  |  | **Posterior probablities** | | |  |  |
|  |  |  |  | From |  |  |  |  |  |  | From |  |  |  |
|  |  |  | **rC** | **rTPJ** | **rPRE** |  |  |  |  | **rC** | **rTPJ** | **rPRE** |  |  |
|  |  | **rC** |  | 0.93 | 1.05 |  |  |  | **rC** |  | 0.65 | 0.67 |  |  |
| **F** | To | **rTPJ** | 1.03 |  | 1.00 |  |  | To | **rTPJ** | 0.74 |  | 0.70 |  |  |
|  |  | **rRPE** | 1.02 | 0.99 |  |  |  |  | **rRPE** | 0.74 | 0.67 |  |  |  |
|  |  |  |  |  |  |  |  |  |  |  |  |  |  |  |
|  |  |  |  |  |  |  |  |  |  |  |  |  |  |  |
|  |  |  |  | From |  |  |  |  |  |  | From |  |  |  |
|  |  |  | **rC** | **rTPJ** | **rPRE** |  |  |  |  | **rC** | **rTPJ** | **rPRE** |  |  |
|  |  | rC |  | 0.93 | 1.03 |  |  |  | **rC** |  | 0.71 | 0.66 |  |  |
| **B** | To | rTPJ | 0.98 |  | 0.99 |  |  | To | **rTPJ** | 0.79 |  | 0.69 |  |  |
|  |  | rRPE | 0.98 | 0.84 |  |  |  |  | **rRPE** | 0.75 | 0.75 |  |  |  |
|  |  |  |  |  |  |  |  |  |  |  |  |  |  |  |
|  |  |  |  |  |  |  |  |  |  |  |  |  |  |  |
|  |  |  |  | From |  |  |  |  |  |  | From |  |  |  |
|  |  |  | **rC** | **rTPJ** | **rPRE** |  |  |  |  | **rC** | **rTPJ** | **rPRE** |  |  |
|  |  | **rC** |  | 1.05 | 0.94 |  |  |  | **rC** |  | 0.68 | 0.61 |  |  |
| **FB** | To | **rTPJ** | 1.01 |  | 1.09 |  |  | To | **rTPJ** | 0.82 |  | 0.72 |  |  |
|  |  | **rRPE** | 1.04 | 0.86 |  |  |  |  | **rRPE** | 0.70 | 0.74 |  |  |  |
|  |  |  |  |  |  |  |  |  |  |  |  |  |  |  |
